# Severe Class III Lupus Nephritis With Concurrent Thrombotic Microangiopathy and Suspected Atypical Hemolytic Uremic Syndrome Requiring Complement Blockade: A Complex Multisystem Presentation

**DOI:** 10.7759/cureus.111765

**Published:** 2026-06-29

**Authors:** Nishma Pokharel, Arsalan Alvi, Alejandro Best

**Affiliations:** 1 Department of Internal Medicine, MercyOne Medical Center, Des Moines, USA; 2 Department of Nephrology, MercyOne Medical Center, Des Moines, USA; 3 Department of Pathology and Laboratory Medicine, Arkana Laboratories, Little Rock, USA

**Keywords:** atypical hus, eculizumab, lupus nephritis, sle, thrombotic microangiopathy

## Abstract

Systemic lupus erythematosus (SLE) is frequently complicated by lupus nephritis (LN), but the coexistence of thrombotic microangiopathy (TMA) represents a rare and severe manifestation associated with poor renal outcomes. Differentiating between SLE-associated TMA and primary complement-mediated atypical hemolytic uremic syndrome is diagnostically challenging but critical, as it dictates the use of targeted therapies such as terminal complement inhibitors. A 36-year-old female with SLE, lost to follow-up for two years, presented with fatigue, seizures, and anuric renal failure. Laboratory studies revealed severe bicytopenia, metabolic acidosis, and hemolysis with normal ADAMTS13 activity. A renal biopsy confirmed International Society of Nephrology/Renal Pathology Society Class III LN with prominent superimposed TMA. Despite intensive management with high-dose steroids, plasma exchange, and hemodialysis, the patient’s refractory state necessitated the initiation of eculizumab. The clinical course was further complicated by a positive direct anti-globulin test (C3 positive/IgG negative), requiring intravenous immunoglobulin for a suspected secondary immune-mediated anemia. This case illustrates the diagnostic complexity of LN-TMA and the therapeutic necessity of a multidisciplinary approach. It underscores the importance of early renal biopsy and the timely utilization of complement inhibition in refractory cases to mitigate irreversible renal damage.

## Introduction

Thrombotic microangiopathy (TMA) in the setting of systemic lupus erythematosus (SLE) represents a critical diagnostic and therapeutic crossroads characterized by profound diagnostic overlap. In patients with lupus nephritis (LN), TMA is not merely a histopathologic bystander but a driver of rapid, often irreversible, renal decline. Distinguishing between SLE-related TMA, atypical hemolytic uremic syndrome (aHUS), and thrombotic thrombocytopenic purpura (TTP) remains a formidable challenge. These entities frequently share a common clinical triad of microangiopathic hemolytic anemia, thrombocytopenia, and acute kidney injury (AKI), masking the underlying driver of endothelial damage [1,2]. However, central to this overlap is the alternative complement pathway. While SLE is classically associated with the classical pathway (C3 and C4 consumption), LN-associated TMA frequently involves uncontrolled activation or "over-activation" of the alternative and lectin pathways. This dysregulation, whether driven by genetic mutations in complement factors or acquired autoantibodies, results in a continuous loop of endothelial injury and microthrombi formation. Clinically, this distinction is vital: the activation of the alternative pathway signifies a shift from purely immune-complex-mediated inflammation to a self-sustaining thrombotic state, where standard lupus induction therapy may fail to halt the process [2-4].

TMA is identified in approximately 3-24% of LN biopsies, and its presence serves as a devastating prognostic marker [1,2]. Patients with LN-TMA overlap face a disproportionately high risk of poor renal recovery, with studies indicating a 40-60% higher risk of progressing to end-stage kidney disease compared to those with isolated LN. This association with dialysis dependence and permanent cortical damage underscores the necessity for rapid, aggressive intervention [3,4].

Given the failure of traditional immunosuppression to address this microvascular crisis, the role of complement blockade has emerged as a pivotal therapeutic strategy. The use of eculizumab addresses the terminal complement cascade that links SLE-induced damage to the aHUS-like phenotype [4].

## Case presentation

A 36-year-old woman with known SLE, previously followed at a university hospital but lost to follow-up for two years due to social stressors, presented to an outside emergency department with several days of worsening fatigue, diffuse body aches, mild abdominal pain, and two self-reported seizures. She had reportedly well-controlled SLE with hydroxychloroquine and mycophenolate (reported non-compliance) until 2020. She had been off therapy since 2020.

On arrival, the patient’s vitals were significant for elevated systolic blood pressure of around 190s. Laboratory testing was consistent with anuric renal failure. Nephrology was consulted for acute kidney injury and anuria, and the patient was initiated on hemodialysis. At presentation, differential diagnoses included TTP, complement-mediated aHUS, antiphospholipid syndrome (APS)-associated nephropathy, malignant hypertension-related TMA, drug-induced TMA, and lupus flare with secondary microangiopathy. ADAMTS13 activity was not severely reduced, reducing the likelihood of TTP. APS testing was negative, and there was no history of causative medication exposure. Renal biopsy performed on hospital day two demonstrated TMA, focal International Society of Nephrology/Renal Pathology Society Class III LN, and collapsing glomerulopathy.

The modified National Institutes of Health lupus nephritis activity score was 3/24, with a chronicity score of 8/12, indicating TMA as the predominant contributor to renal dysfunction superimposed on pre-existing LN. Urinalysis was positive for proteinuria with no hematuria. Hematologic evaluation revealed evidence of hemolysis with low haptoglobin <10, increased schistocytes and microspherocytes, low complement levels, elevated soluble C5b-9, and a direct antiglobulin test negative for IgG but positive for C3, findings that supported complement-mediated TMA and made warm autoimmune hemolytic anemia less likely. Additional serologic testing showed perinuclear antineutrophil cytoplasmic antibody positivity with negative myeloperoxidase antibodies and elevated ADAMTS13 activity, effectively ruling out TTP as the primary cause of the TMA. The patient experienced two generalized tonic-clonic seizures at presentation. Electroencephalography was unremarkable, and brain imaging did not demonstrate acute structural abnormalities. The seizures were attributed to severe metabolic derangements and uremia in the context of acute renal failure (Table 1).

**Table 1:** Hematologic and renal parameters on admission.

Parameter	Unit	Baseline (July 2023)	On admission (Day 0)	Reference range
Complete blood count				
Hemoglobin	g/dL	-	6.9	13.5–17.5
White blood cell count	/µL	-	11,000	4,500–11,000
Platelets	/µL	-	40,000	150–450 ×10³/μL
Metabolic panel				
Creatinine	mg/dL	0.91	5.22	0.7–1.3
Bicarbonate	mmol/L	-	12	22–29
Hemolysis and immunology				
Haptoglobin	mg/dL	-	10 (low)	30–200
C3 complement	mg/dL	-	39 (low)	90–180
C4 complement	mg/dL	-	4 (low)	10–40
sC5b-9	ng/mL	-	315 (elevated)	≤250
ADAMTS13 activity		-	Elevated	>10%
DAT (direct Coombs)		-	IgG (-), C3 (+)	Negative

**Figure 1:**
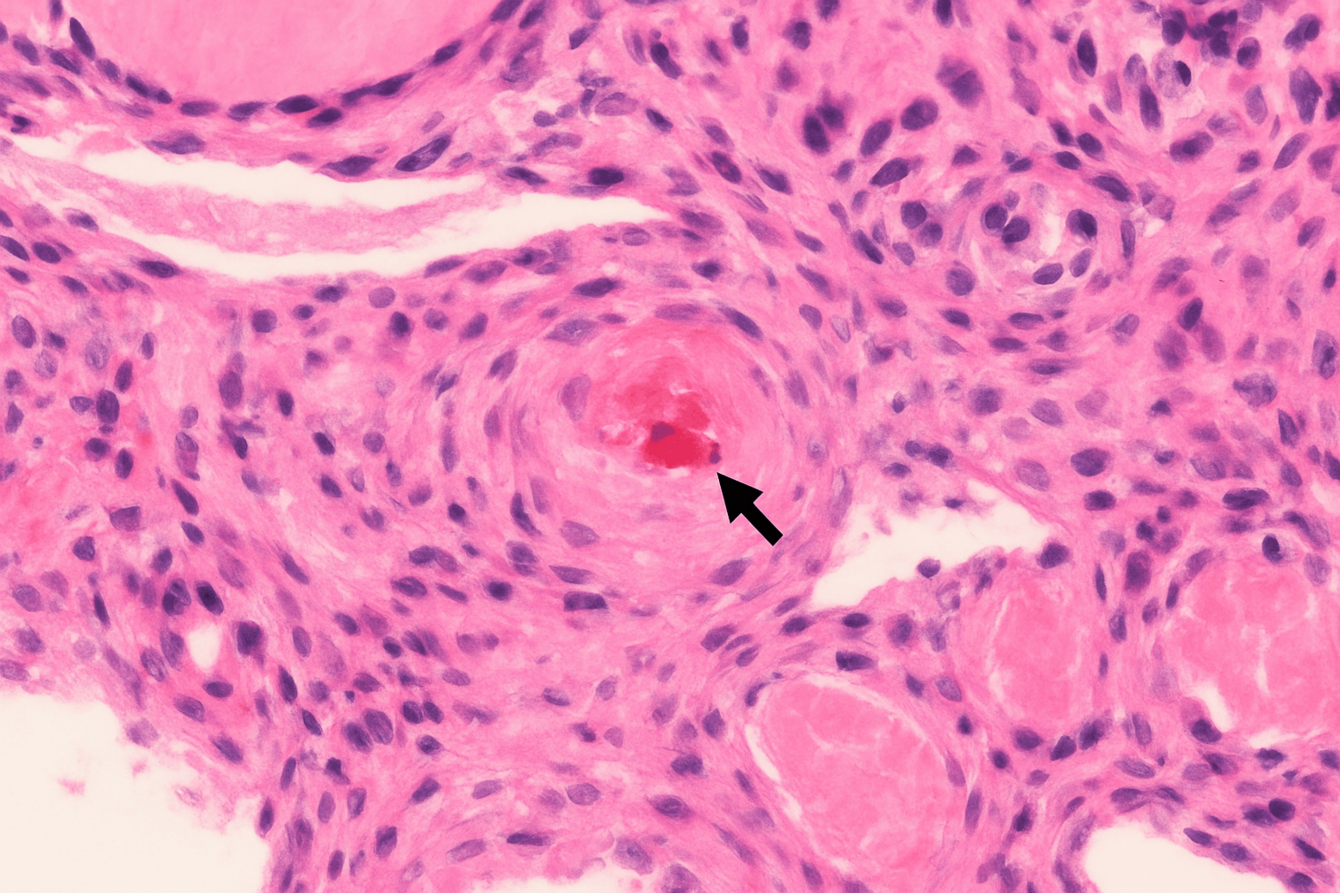
Renal biopsy showing fibrin-rich thrombi within arterioles consistent with thrombotic microangiopathy.

Figure 1 demonstrates microvascular thrombi, a classic histopathologic feature of TMA. In this case, TMA appeared to be the predominant contributor to the patient’s renal dysfunction, superimposed on pre-existing LN.

Given severe TMA and concern for complement-mediated aHUS in the setting of SLE, the patient was started on high-dose intravenous methylprednisolone and daily plasma exchange. Due to anticipated delays in obtaining complement inhibitor therapy through a tertiary center, eculizumab was arranged and administered inpatient, after which she was transitioned to prednisone 60 mg daily and continued hydroxychloroquine 200 mg daily.

Due to infection risk associated with complement blockade, prophylaxis with atovaquone and penicillin VK was initiated, and MenACWY and MenB vaccines were administered per eculizumab guidelines. Her course was further complicated by a direct antiglobulin test pattern positive for C3 alone with negative IgG. Hematology elected to administer intravenous immunoglobulin empirically to address a possible immune-mediated component to her anemia.

Despite therapy, she remained dialysis-dependent. Due to difficulty arranging her next scheduled eculizumab dose locally, the patient was transferred to a higher facility for continued management on hospital day 12.

## Discussion

LN complicated by TMA is a rare but serious manifestation of SLE, associated with rapid renal deterioration and poor prognosis [4-6]. TMA in SLE may occur due to immune complex-mediated endothelial injury, antiphospholipid antibodies, malignant hypertension, or complement dysregulation [4-12].

Distinguishing primary complement-mediated aHUS from lupus-associated secondary TMA remains inherently challenging. Active LN alone can induce endothelial injury and complement activation sufficient to produce a secondary TMA phenotype without underlying genetic dysregulation. In our case, although aHUS was favored due to persistent hemolysis despite plasma exchange and normal ADAMTS13 activity, we must acknowledge that a definitive distinction between primary aHUS and severe SLE-associated TMA may not be possible. Diagnostic labeling often relies on clinical probability rather than molecular confirmation. While APS nephropathy was considered, the absence of antiphospholipid antibodies, assessed during the acute phase, reduced its likelihood, though the limitation of single-point testing in the setting of acute consumption is noted [6]. Furthermore, the lack of complement genetic testing represents a limitation in definitively confirming an inherited pathway abnormality, which is often the gold standard for an aHUS diagnosis.

A significant feature in our patient’s renal biopsy was the presence of collapsing glomerulopathy. This finding is a potent independent predictor of poor renal outcomes and likely contributed significantly to the patient’s progression to dialysis. When correlated with the high chronicity scores and prominent vascular injury observed on pathology, these features provide a clearer explanation for the lack of renal recovery despite intensive therapy. The irreversible nature of the collapsing features and established chronic damage often supersedes the benefits of late-stage interventions.

The role of complement-mediated aHUS in SLE-associated TMA has been increasingly recognized, and complement inhibition with eculizumab has demonstrated improved outcomes in some cases [8-13]. However, the evidence for eculizumab in SLE-associated TMA is variable. While our patient received early complement blockade due to laboratory evidence of hemolysis, the response to such therapy is not universal. Predictors of response remain poorly defined, and many SLE-associated TMAs do not respond if the underlying drivers are predominantly inflammatory or if irreversible vascular damage has already occurred. This case illustrates the uncertainty surrounding the timing and efficacy of complement blockade in the setting of multisystem autoimmune flares.

Overlapping hematologic abnormalities, such as warm autoimmune hemolytic anemia, are occasionally observed in SLE. Our patient’s positive direct antiglobulin test, specifically C3+, emphasizes the complex overlap between complement-mediated hemolysis and immune hemolysis [14]. This pattern prompted the use of intravenous immunoglobulin to address potential immune-mediated destruction, highlighting the need for a multidisciplinary approach to differentiate between mechanical fragmentation and antibody-mediated processes.

Compared to published cases by Song et al. and Tektonidou et al., our patient presented with a similarly severe clinical triad of anemia, thrombocytopenia, and elevated creatinine [6,7]. However, the addition of collapsing glomerulopathy and the lack of response to eculizumab sets this case apart from more favorable reports, aligning it with high-risk cohorts where chronic biopsy changes dictate the long-term prognosis regardless of the intensity of immunosuppression.

## Conclusions

This case illustrates the significant challenges in managing LN when complicated by TMA. While we suspected complement-mediated injury, the clinical overlap between SLE and aHUS makes a definitive diagnosis difficult, even with early intervention. In this instance, the initiation of complement blockade did not lead to renal recovery. This lack of response, despite timely treatment, likely reflects the severity of the underlying biopsy findings, specifically the collapsing glomerulopathy and irreversible vascular damage. Ultimately, this case serves as a reminder that even with a multidisciplinary approach and early therapy, the prognosis for these patients remains guarded, and treatment outcomes are highly variable.
